# Physicochemical Properties and Inductive Effect of Calcium Strontium Silicate on the Differentiation of Human Dental Pulp Stem Cells for Vital Pulp Therapies: An In Vitro Study

**DOI:** 10.3390/ma15175854

**Published:** 2022-08-25

**Authors:** Mohamed Mahmoud Abdalla, Christie Y. K. Lung, Mohammed Nadeem Bijle, Cynthia Kar Yung Yiu

**Affiliations:** 1 Paediatric Dentistry, Faculty of Dentistry, The University of Hong Kong, Hong Kong, China; 2 Dental Biomaterials, Faculty of Dental Medicine, Al-Azhar University, Cairo 11651, Egypt; 3 Dental Materials Science, Applied Oral Sciences, Faculty of Dentistry, The University of Hong Kong, Hong Kong, China; 4 Paediatric Dentistry, Department of Clinical Sciences, College of Dentistry, Ajman University, Ajman P.O. Box 346, United Arab Emirates

**Keywords:** calcium strontium silicate, setting time, bioactivity, dental pulp stem cells, sol–gel, differentiation

## Abstract

The development of biomaterials that exhibit profound bioactivity and stimulate stem cell differentiation is imperative for the success and prognosis of vital pulp therapies. The objectives were to (1) synthesize calcium strontium silicate (CSR) ceramic through the sol–gel process (2) investigate its physicochemical properties, bioactivity, cytocompatibility, and its stimulatory effect on the differentiation of human dental pulp stem cells (HDPSC). Calcium silicate (CS) and calcium strontium silicate (CSR) were synthesized by the sol–gel method and characterized by x-ray diffraction (XRD). Setting time, compressive strength, and pH were measured. The in vitro apatite formation was evaluated by SEM-EDX and FTIR. The NIH/3T3 cell viability was assessed using an MTT assay. The differentiation of HDPSC was evaluated using alkaline phosphatase activity (ALP), and Alizarin red staining (ARS). Ion release of Ca, Sr, and Si was measured using inductive coupled plasma optical emission spectroscopy (ICP-OES). XRD showed the synthesis of (CaSrSiO_4_). The initial and final setting times were significantly shorter in CSR (5 ± 0.75 min, 29 ± 1.9 min) than in CS (8 ± 0.77 min, 31 ± 1.39 min), respectively (*p* < 0.05). No significant difference in compressive strength was found between CS and CSR (*p* > 0.05). CSR demonstrated higher apatite formation and cell viability than CS. The ALP activity was significantly higher in CSR 1.16 ± 0.12 than CS 0.92 ± 0.15 after 14 d of culture (*p* < 0.05). ARS showed higher mineralization in CSR than CS after 14 and 21 d culture times. CSR revealed enhanced differentiation of HDPSC, physicochemical properties, and bioactivity compared to CS.

## 1. Introduction

In recent years, there has been an increasing interest in bioactive materials and regenerative dentistry [1,2], which demands the development of biomaterials that exhibit profound bioactivity, biocompatibility, and odontogenic activity [3]. Since most biomaterials have direct or indirect contact with vital tissues in procedures such as direct/indirect pulp capping, pulpotomy, perforation repair, apexification, apexogenesis, and regenerative endodontics, they must therefore possess biological properties that stimulate tissue regeneration and cellular differentiation [4].

The human dental pulp is a complex tissue of blood vessels, nerve fibers, connective tissue, and mesenchymal stem cells, which provides vascular and neural supplies to the tooth structure and supports the repair of tooth dentin [5,6]. Thus, preservation of pulp vitality is critical to the effectiveness of vital pulp therapies.

Calcium silicate (CS)-based ceramics (CaSiO_3_, di-calcium silicate, and tri-calcium silicate) have received considerable interest owing to their evident bioactivity and biocompatibility. Calcium silicate-based materials have been widely used as root-end filling materials, in vital/non-vital pulp therapies, and extending their applications to regenerative endodontics, implant coatings, and orthopedic treatments [7,8]. However, the increased degradation potential of calcium silicate, high pH levels that retard cellular growth, insufficient stimulation of cellular differentiation, low chemical stability, and inherent radiopacity [3,9,10] remain to be significant problems with their utilization. Accordingly, it is imperative to develop novel biomaterials with improved bioactivity and biocompatibility which can overcome the drawbacks of calcium silicate ceramics while enhancing their odonto/osteogenic potential.

The sol–gel synthesis of calcium silicate-based ceramics is one successful method for optimizing their physicochemical and biological properties [11]. The synthesized powders are characterized by immense purity, homogeneity, and bioactivity [12,13]. Additionally, doping calcium silicate networks with inorganic ions has been reported to overcome the drawbacks of calcium silicate ceramics, making them more suitable for dental and biomedical applications [10,14,15]. The incorporation of inorganic divalent cations (i.e., Sr, Zn, Mg, Cu, Co) into calcium silicates improved their biological performance, handling quality, bioactivities, chemical stability, and antibacterial activity [10,15].

One interesting alkaline earth metal is strontium (Sr). Sr is found in the skeleton and in minute amounts in teeth [16]. Sr has attracted researchers’ attention for its significant effects on bone deposition and inhibition of bone resorption. It enhances bone regeneration by activating osteoblasts and minimizes the incidence of bone fractures in patients with osteoporosis [17]. In addition, strontium was also found to stimulate the odontogenic differentiation of human dental pulp stem cells (HDPSC) [16]. It has remarkable radiopacity, which can potentially improve the radiopacity of calcium silicates [3,18]. Furthermore, Sr has been reported to alleviate dentine hypersensitivity [19] when incorporated in dentifrices, which effectuates by occluding dentinal tubules [16,20]. Additionally, Sr has demonstrated a synergistic effect with fluorides to prevent dental caries [21,22]. Taken all together, combining calcium silicate and Sr would develop a promising multipurpose biomaterial for various dental applications.

Several studies have incorporated Sr in different ceramics, such as Sr-doped calcium silicate (CaSiO_3_) for plasma-sprayed implant coatings [21], Sr-containing bioactive glass [23], and strontium-doped calcium silicate/gypsum bioactive bone cement [24,25]. All these formulations demonstrated enhanced physical and biological properties after Sr incorporation when compared to the respective controls. A recent study published by Pelepenko et al. [26] investigated the long-term inflammatory response to strontium-substituted ceramics and concluded that strontium showed negligible local or systemic adverse effects. Most of the previously reported in vitro and in vivo studies on strontium substituted ceramics investigated their effect on bone repair, healing, and osteogenesis, whereas limited literature research is available on the effect of strontium-doped bioceramics on the differentiation of HDPSC.

Human dental pulp stem cells are multipotent stem cells existing in the pulps of teeth. They are rapidly proliferating stem cells and can differentiate into odontoblast-like cells that form reparative dentin and protect the pulp’s health and vitality [6,27]. The differentiation of HDPSC into mature mineralizing linage is a key factor in the success of vital pulp therapies [28]. Hence, the pulp-capping biomaterial should possess the potential to stimulate HDPSC differentiation. So far, no study has investigated the effect of calcium strontium silicate on the differentiation of human dental pulp stem cells.

Therefore, the present study aimed primarily to synthesize self-setting nanosized calcium strontium silicate (CSR) bioceramics using the sol–gel method and investigate its physicochemical properties, bioactivity, cytocompatibility, and its stimulatory effect on the differentiation and mineralization of HDPSC. The null hypothesis tested was that there was no statistically significant difference in physicochemical properties, bioactivity, cytocompatibility, and stimulatory effect on the differentiation and mineralization of HDPSC between CS and CSR.

## 2. Materials and Methods

### 2.1. Synthesis Procedures of the Experimental Cements

CS and CSR were prepared by the sol–gel method as per our earlier study [10] with some modifications. In brief, tetraethyl orthosilicate (Si(OC_2_H_5_)_4_; TEOS, Sigma-Aldrich, 98.0%, St. Louis, MO, USA) was added to 4 mL deionized water (DIW) containing 40 µL of HNO_3_ (69%, VWR PROLABO CHEMICALS, Radnor, PA, USA). Then Ca(NO_3_)_2_⋅4H_2_O and Sr(NO_3_)_2_ (Sigma-Aldrich, 99.0%, St. Louis, MO, USA) were added, respectively, with continuous mixing for 1 h. The molar ratio of Ca:Sr:Si was 3:1:1.04. The mixture was heated at 60 °C for 24 h and dried at 120 °C for 48 h. The dry gel was sintered at 1450 °C for 5 h. Finally, powders were refined in a ball mill and sieved. CS was prepared following the same procedures with a Ca:Si molar ratio of 3:1 and served as the control group.

### 2.2. Characterization of the Synthesized Powder

The powders were put into a 2 cm × 2 cm × 0.05 cm chamber of a glass slide. The measurements were performed using an X-ray diffractometer (XRD, Rigaku SmartLab 9 kW, Tokyo, Japan) equipped with a copper rotating anode (K 1 1.541, Ka2 1.544) at 200 mA current and 45 kV voltage. Diffraction signals were filtered with a K_β_ Nickel filter, and data were collected with a scintillation counter detector. The scanning range was set to 5–60° and the scanning speed was 2°/min. Phase identification was matched using JADE pattern identification software (JADE v. 6.5, Livermore, CA, USA).

### 2.3. Samples Preparation

The following mixing steps were set to prepare all the specimens, with different shapes and sizes of the molds used for different experiments. A cement to liquid (DIW) ratio of 3:1 was used in mixing the synthesized cement. After which, they were kept in an incubator at 37 °C and 95 ± 5% humidity for 24 h for the hydration and setting of the materials prior to testing.

### 2.4. ZetaView Particle Size Analyzer

The ZetaView analyzer (Particle Metrix, PMX 120-Z, GMBH, Inning am Ammersee, Germany) was used to measure the particle size of the synthesized powders. Following instrument calibration, the experimental powder was diluted in DIW, and 1 mL was injected into the instrument chamber for automated measurements at eleven distinct positions in the cell channel.

### 2.5. Setting Time

Measuring the setting time was completed using the Gilmore needle indentation method, which was modified slightly from ASTM C266-15. Disc-like specimens (n = 6) from each individual material were mixed as mentioned in Section 2.3 using plastic molds of 10 ± 0.1 mm in diameter and 2 ± 0.1 mm thickness. The Gilmore needle (diameter: 2 ± 0.1 mm, and a weight of 113.5 ± 5 g) was gently dragged down on the specimen surface every 60 s for the initial setting time until no visible indentation was detected on the surface. To determine the final setting time, a Gilmore needle (453.5 ± 5 g in weight) was lowered onto the sample surface every 180 s until no further indentations were visible. The final setting time was considered from the beginning of mixing until no indentation was noticed on the surface of the specimens.

### 2.6. Compressive Strength Test

Compressive strength measurements were performed following the protocol recommended by ISO 9917-1. Six specimens (diameter = 3 mm, height = 6 mm) cylindrical in shape were prepared from each group and subjected to compressive strength testing (after 7 days of incubation at 37 °C and 95 ± 5% humidity) on the universal testing machine (ElectroPuls™ E3000, Instron, Norwood, MA, USA) at 1.0 mm/min crosshead speed till fracture occurred. The maximum force was measured, and the compressive strength was computed in megapascals (MPa) as follows:

σ = 4F/πd^2^

where σ refers to the compressive strength (MPa); F, the maximum force before failure; and d, is the diameter of the specimen in mm.

### 2.7. pH Measurement

For pH measurements, 3 discs of 10 ± 0.1 mm in diameter and 2 ± 0.5 mm in height were prepared from individual materials and incubated for 24 h at 37 °C and 95 ± 5% relative humidity. After that, each disc was placed in a plastic tube containing 15 mL of Hank’s balanced solution (HBSS). Using a calibrated pH meter, the pH was measured immediately (initial), after 24 h, and after 72 h of immersion in HBSS. HBSS was used as a control. The pH meter was calibrated using three standard calibrating solutions (pH 4.01, 7.01, and 10.00) prior to each test.

### 2.8. In Vitro Apatite Formation and Characterization of the Formed Apatite Layer

The in vitro apatite forming ability of the fabricated cement was assessed by immersion in phosphate-buffered saline (PBS, 2.7 mmol L^−1^ KCl, 136.4 mmol L^−1^ NaCl, 8.2 mmol L^−1^ NaH_2_PO_4_, and 1.25 mmol L^−1^ KH_2_PO_4_, (Sigma-Aldrich, St. Louis, MO, USA) in 1 L of DIW at pH 7.4 free of Ca and Mg for 1, 7 and 28 days [29]. Three-disc specimens 5 mm in diameter and 2 mm in thickness were immersed in PBS which was replaced every 7 days in the case of the 28-days group. At the end of each immersion time point, the specimens were gently washed with DIW water before being analyzed.

To further investigate the formed apatite-like layer, the top layer of the discs was gently scraped off and characterized using attenuated total reflection/Fourier-transformed infrared (ATR-FTIR, L160000 Spectrum-Two spectrophotometer instrument. PerkinElmer Inc. Waltham, MA, USA) at 400–4000 cm^−1^ wavenumber range. The Ca/P molar ratio was also estimated using SEM-EDX (Model 550i, IXRF Systems, Austin, TX, USA) with an accelerating voltage of 15 kV at 1000× magnification using SEM (SU1510, Hitachi, Ibaraki, Japan) at three randomly selected areas on the specimens. Finally, the specimens were inspected under the SEM after sputter-coating with 80% platinum and 20% palladium for the formation of apatite-like crystals on their surface.

### 2.9. Cell Viability and MTT Assay

NIH/3T3 mouse fibroblasts (Central Research Laboratory, Faculty of Dentistry, The University of Hong Kong, Hong Kong, China) were grown in Dulbecco modified Eagle medium (DMEM, GIBCO BRL, Gaithersburg, MD, USA) supplemented with 10% fetal bovine serum (FBS, Gibco, Gaithersburg, MD, USA), streptomycin (50 g/mL), and a 1% antibiotic at 37 °C, 100% humidity, 95% air, and 5% CO_2_. The cultivated cells were exposed to the materials’ extracts. Regarding the preparation of extracts: two discs from individual materials (6 mm in diameter and 2 mm in thickness) were prepared under controlled aseptic conditions and sterilized using ultraviolet light for 20 min. The specimens were placed into sterile vials containing 1 mL of DMEM (served as the extraction medium) and incubated at 37 °C for 3 days to leach the material components into the medium, and then filtered through a 0.22 µm filter (Acrodisc syringe filter, Pall Corp., New York, NY, USA). The proportion of the area of discs to the volume of the medium was 0.95 cm^2^/mL (International Standard Organization 10993-5). Two dilutions (1:1 dilution and 1:2 dilution) were prepared using DMEM. The cells were seeded at a concentration of 4 × 10^4^ cells/well in a 96-well plate and incubated for 24 h. Following medium aspiration, 100 µL of each concentration was added to the seeded cells and incubated for 1 and 3 days, respectively. Cells incubated in DMEM alone (without material extracts) served as the respective control. 

The cell viability was measured using 3-(4,5-dimethylthiazol-2-yl)-2,5-diphenyltetrazolium bromide (MTT). After aspirating all the extracts, each well received ten µL of the prepared MTT stock with 100 µL of DMEM. The cells were incubated at 37 °C for 4 h, then 85 µL of media was removed and 50 µL of dimethyl sulfoxide (DMSO) was added to each well, followed by 10 min of incubation at 37 °C. A spectrophotometer (Spectra max M2E, Molecular devises, San Jose, CA, USA) was used to measure the optical density (OD) at 540 nm. The experiment was conducted three times at three separate occasions.

### 2.10. Differentiation of HDPSC

The differentiation of HDPSC into mature mineralizing cells after exposure to the materials’ extract was evaluated using ARS and ALP activity assays. HDPSC (Central Research Laboratory, Faculty of Dentistry, The University of Hong Kong, Hong Kong, China), previously isolated from freshly extracted human impacted third molar, were cultured in α-minimal essential medium (α-MEM, GIBCO BRL, Gaithersburg, MD, USA) supplemented with 10% FBS (Gibco), streptomycin (50 g/mL), and a 1% antibiotic in a humidified atmosphere containing 10% CO_2_ at 37 °C. The 3rd–5th passages were used for all the experiments. The cells were seeded in a 12-well plate at a density of 1 × 10^5^ cells/well at 37 °C, 100% humidity, 95% air, and 5% CO_2_ for 24 h before they were subjected to the treatment solutions. The material extracts were added to the differentiation medium (DM, containing 10% FBS, 50 mg/mL L-ascorbic acid, 5 mM b-glycerophosphate, and 10 nM dexamethasone). The medium was replaced every 48 h during the designated induction periods. Cells treated with regular α-MEM (RM) only served as the control. All the differentiation assays were performed in triplicate.

#### 2.10.1. Alkaline Phosphatase Activity

The ALP enzymatic activity of the differentiated HDPSC was assessed using a SensoLyte^®^ p-Nitrophenyl phosphate (pNPP) Alkaline Phosphatase colorimetric kit (AnaSpec, Fremont, CA, USA) according to the manufacturer’s instructions. HDPSC (1 × 10^5^ cells/well) were seeded for 3, 7, and 14 days. After every time point, the cells were gently washed twice with 1× assay buffer, followed by the addition of Triton X-100 solution to each well. The attached cells were scraped off, and the cell suspension was collected in microcentrifuge tubes and incubated for 10 min under agitation at 4 °C, with subsequent centrifuging at 2500× *g* for 10 min at 4 °C. The ALP activity was detected by adding fifty μL of the collected supernatants from each sample into a 96-well plate, followed by adding 50 μL of pNPP substrate solution into each well and mixing them gently by shaking the plate for 30 s. After incubation for 60 min at 37 °C, the plate was shaken for 1 min, and the OD was read at 405 nm in a spectrophotometer (Spectra max M2E multi-mode microplate reader, Molecular Devices, Invitrogen, San Jose, CA, USA) [30,31].

#### 2.10.2. Alizarin Red Staining

ARS was used to detect calcium deposits (mineralization) in the culture media. HDPSC (1 × 10^5^ cells/well) were cultured for 14, and 21 days. Subsequently, the cells were washed twice with PBS and fixed with 10% paraformaldehyde at room temperature for 30 min. ARS solution (2% at pH 4.1) was gently applied to each well and incubated for 30 min at room temperature, and then washed away 4 times with DIW. After air drying, the plates were imaged using a scanner (Bio-Rad, GS-800 calibrated imaging densitometer, Hercules, CA, USA). 

To quantify the formed ARS, 1ml of 10% acetic acid was applied to the wells and incubated for 30 min, and then at 85 °C for 10 min. After cooling down, 100 μL from each group was added to a 96-well plate. The OD was read at 405 nm on a plate reader (Spectra max M2E multi-mode microplate reader, Molecular Devices, Invitrogen, San Jose, CA, USA) [32]. 

### 2.11. Ion Release

The ion release of Ca, Sr, and Si at pH 7.0 was measured using ICP-OES (SPECTRO ARCOS, SPECTRO, Kleve, Germany). Six cylindrical specimens (6 mm in height × 3 mm in diameter) from each group were prepared. After soaking for 24 h in 5 mL DIW at pH 7.0, the specimens were moved to new vials with fresh solutions after 7 and 14 days. The standard curves of Ca, Sr, and Si were plotted, followed by measuring Ca, Sr, and Si release in the treatment solutions. The measurements were performed in triplicate. 

### 2.12. Statistical Analysis

The statistical package for the social sciences (SPSS) version 26 (IBM, Armonk, NY, USA) was used to analyze the data. The independent t-test was used to analyze setting time and compressive strength. Two-way ANOVA was used to analyze pH values, ALP, and ARS, where the investigated factors were: factor1: CS, CSR, and controls (groups) and factor 2: (different time points). Cell viability results were analyzed using three-way ANOVA, where the analyzed factors were factor1: CS, CSR, and controls (groups), factor 2: (different time points), and factor3: different dilutions (1:1, 1:2). The Bonferroni post hoc test was used to identify differences between groups following ANOVA, with significance set at *p* < 0.05. 

## 3. Results

### 3.1. Powder Characterization

**Figure 1:**
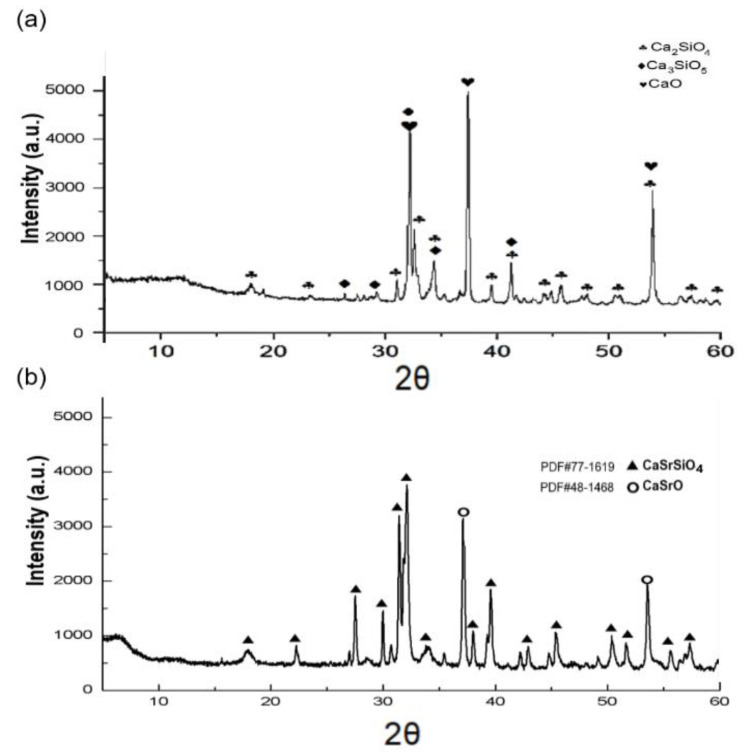
Crystallographic characterization of the synthesized materials. (**a**) XRD patterns of CS exhibiting diffraction peaks of Ca_2_SiO_4_ as the major phase, Ca_3_SiO_5_, and CaO as minor phases. (**b**) XRD phase identification of CSR showing the typical characteristic peaks of CaSrSiO_4_, and two minor peaks of CaSrO.

XRD phase identification of CS and CSR powders is presented in Figure 1. The major constituents of CS were dicalcium silicate (Ca_2_SiO_4_) at 32.2, 34.2, 41.2, and 54.0° 2θ, tricalcium silicate (Ca_3_SiO_5_) at 29.2°, 32.5°, 34.2°, and 41° 2θ, and minor peaks of (CaO) at 32.2, 37.4, and 54° 2θ [10] as presented in Figure 1a. While for CSR, the main detected phase was CaSrSiO_4_ at 32.01°, 31.33°, 31.66°, 31.44°, 39.5°, 37.87°, 29.89°, and 27.40° 2θ which was matched with diffraction card (PDF#77-1619), and minor peaks of CaSrO at 37.07, and 53.43 2θ, matching with the standard card (PDF#48-1468) as shown in Figure 1b.

### 3.2. Particle Size Analysis

**Figure 2:**
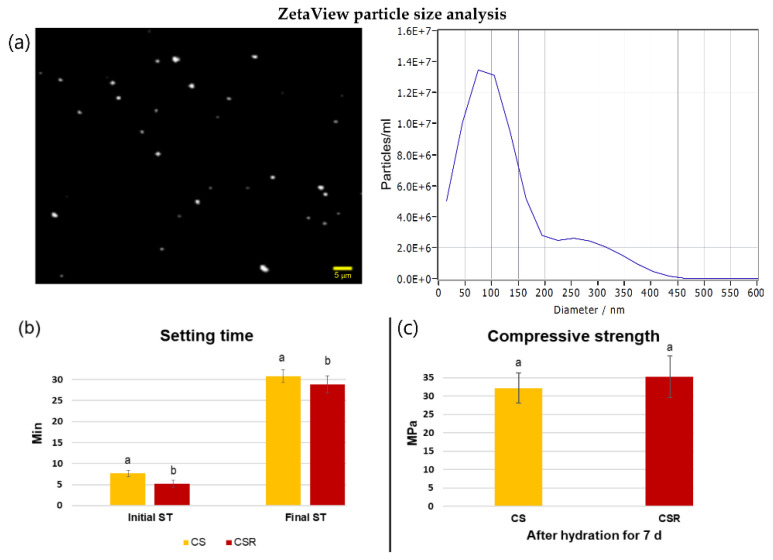
(**a**) ZetaView video and Zeta size distribution of CSR showing the average particles size of 86 nm. Physicochemical properties of the synthesized ceramics. (**b**) The initial and final setting times of CS and CSR. Different lowercase letters indicate significant differences between different groups within each setting time point as analyzed by *t*-test. (**c**) The compressive strength of CS and CSR after hydration for 7 days. No statistically significant difference was found between groups.

The ZetaView particle size analysis showed that the average particle size of the synthesized CSR was 86 nm (Figure 2a).

### 3.3. Setting Time

The initial and final setting times of CS and CSR are presented in Figure 2b. The mean initial and final setting time values of CSR were significantly lower than CS (*p* < 0.05).

### 3.4. Compressive Strength

Figure 2c shows the results of the compressive strength of CS and CSR. The mean compressive strength of CSR was higher than that of CS, but the difference was not statistically significant (*p* = 0.06).

### 3.5. pH Values

The mean pH values of CS and CSR are presented in Table 1. Results of two-way ANOVA showed that factor 1— “groups” was statistically significant (*p* < 0.05). Similarly, factor 2— “time-points” was statistically significant (*p* < 0.05). Additionally, the interaction between the two factors was statistically significant (*p* < 0.05). The estimated pH of CS and CSR were significantly higher than the respective control at all time points (*p* < 0.05). CSR pH was significantly lower than CS at all time points (*p* < 0.05). No significant difference was found in the pH of the control group at all time points (*p* > 0.05), while the pH of CS was significantly different at all time points (*p* < 0.05). CSR showed no significant difference in pH between the 24 h and 72 h time points (*p* > 0.05), while the pH of CSR at both time points (24 and 72 h) was significantly higher than at the initial time point (*p* < 0.05).

### 3.6. In Vitro Apatite Formation

#### 3.6.1. SEM Observation

**Figure 3:**
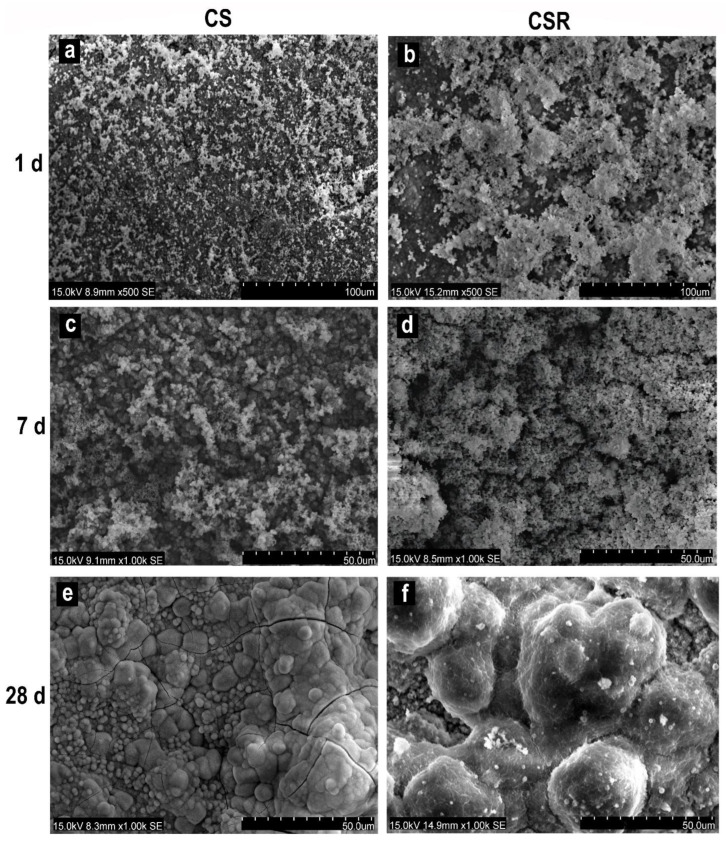
SEM images of CS after immersion in PBS for 1 d (**a**), 7 d (**c**), and 28 d (**e**) showing the deposition of apatite-like layer on the material surface. (**b**,**d**,**f**) are SEM images of CSR after immersion in PBS for 1 d, 7 d, and 28 d, respectively, which show marked increase in the deposited apatite-like layer than in CS at all time points.

After 1 day of immersion in PBS, clusters of apatite spherulites were deposited on the specimens’ surfaces. The formed apatite particles on CSR (Figure 3b) were greater in amount than on CS (Figure 3a). After 7 days, there was a marked increase in the amount and size of the deposited apatite particles on CSR (Figure 3d) than in CS (Figure 3c). After 28 days of PBS immersion, a continuous layer of apatite crystals was observed on the surfaces of both CS and CSR (Figure 3e,f).

#### 3.6.2. FTIR and EDX Analysis

**Figure 4:**
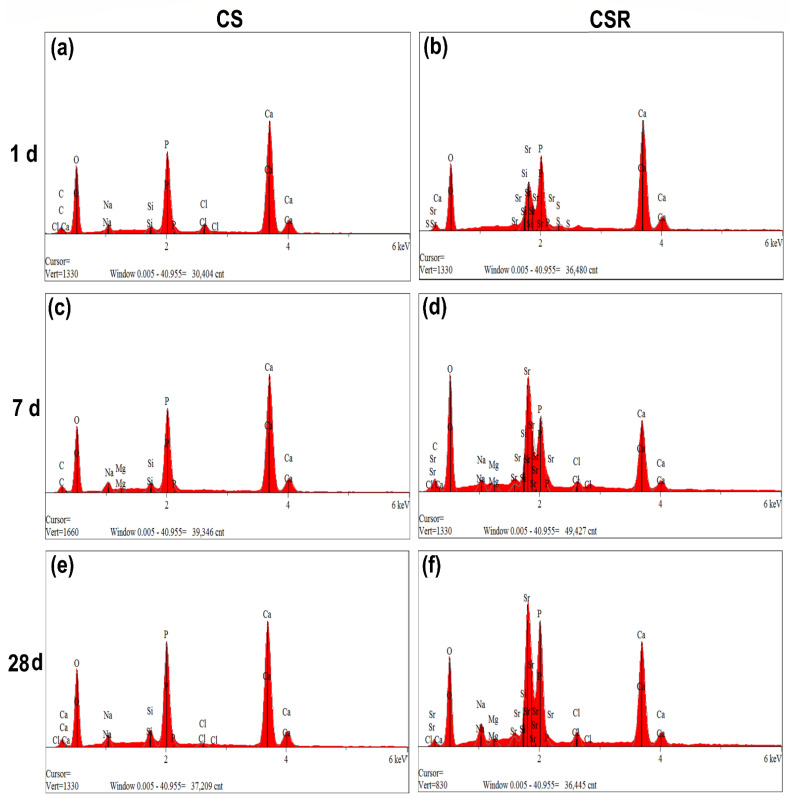
EDX spectra of Ca and P in CS after immersion in PBS for 1 d (**a**), 7 d (**c**), and 28 d (**e**) demonstrate gradual increase in P peak, indicating the formation of apatite-like crystals. Figure 4 (**b**,**d**,**f**) show EDX spectra of Ca and P in CSR which also demonstrated gradual increase in P peak as an indicator for apatite crystals deposition. Sr peak was detected in CSR only.

EDX elemental analysis showed intense peaks of Ca and P at varying degrees among the specimens, in addition to a Sr peak in CSR only, Figure 4a–f. The estimated Ca to P ratio of all the specimens after 28 days in PBS varied from 1.47 to 1.7, indicating the formation of hydroxyapatite-like crystals that was confirmed by FTIR analysis.

**Figure 5:**
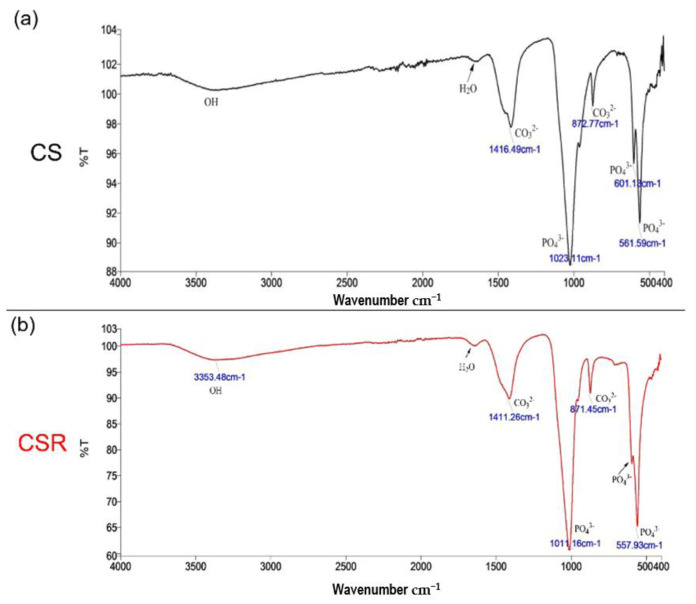
FTIR spectra of Cs (**a**) and CSR (**b**) after 28 d of immersion in PBS. A band split at around 525–650 cm^−1^ and 1070 cm^−1^ which are characteristic indicators of apatite crystals formation.

Figure 5 shows the FTIR spectra of CS (a) and CSR (b) after 28 d of immersion in PBS. For CS, the bands at 561 cm^−1^, 601 cm^−1^, and 1023 cm^−1^ correspond to the PO_4_^3−^ group. The peaks at 872 cm^−1^ and 1416 cm^−1^ correspond to the CO_3_^2−^ group. For CSR, the split band at 567 cm^−1^ corresponds to the PO_4_^3−^ group. The peaks at 871 cm^−1^ and 1411 cm^−1^ correspond to CO_3_^2−^ group. These are characteristic bands indicating hydroxyapatite (HAP) crystal formation. 

### 3.7. Cell Viability

**Figure 6:**
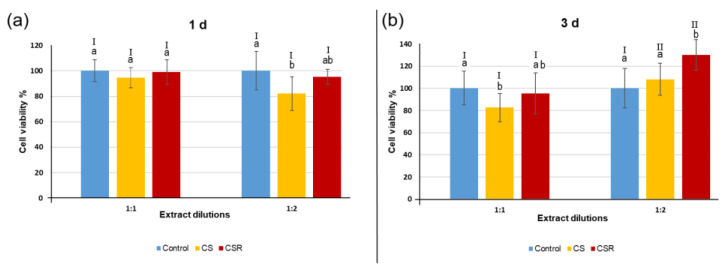
Cell viability results of CS and CSR after incubation with the materials’ extracts for 1 d (**a**) and 3 days (**b**) at two different dilutions 1:1 and 1:2. Different lowercase letters indicate significant difference between groups within each dilution, *p* < 0.05, while Roman numbers I, II indicate difference between dilutions of each group within each time point, *p* < 0.05.

The MTT assay results are shown in Figure 6. Three-way ANOVA analysis showed that all the three factors: 1— “groups”, 2— “time-points”, and 3— “different dilutions” were statistically significant (*p* < 0.05). Additionally, the interaction between the three factors was statistically significant (*p* < 0.05). No significant difference in cell viability was found between the CSR group and control (*p* > 0.05), except at 3 days with 1:2 dilution, where CSR demonstrated significantly higher cell viability than the control group and CS (*p* < 0.05). CS presented significantly lower cell viability than the control group with a 1:2 dilution at 1-day culture and a 1:1 dilution at 3-day culture (*p* < 0.05). Regarding dilutions factor, no significant difference was found between 1:1 dilution and 1:2 dilution at 1-day culture (*p* > 0.05), while CS and CSR showed significantly higher cell viability at 1:2 dilution than at 1:1 dilution at 3-day culture (*p* < 0.05).

### 3.8. HDPSC Differentiation Assays

#### 3.8.1. Alkaline Phosphatase Activity

**Figure 7:**
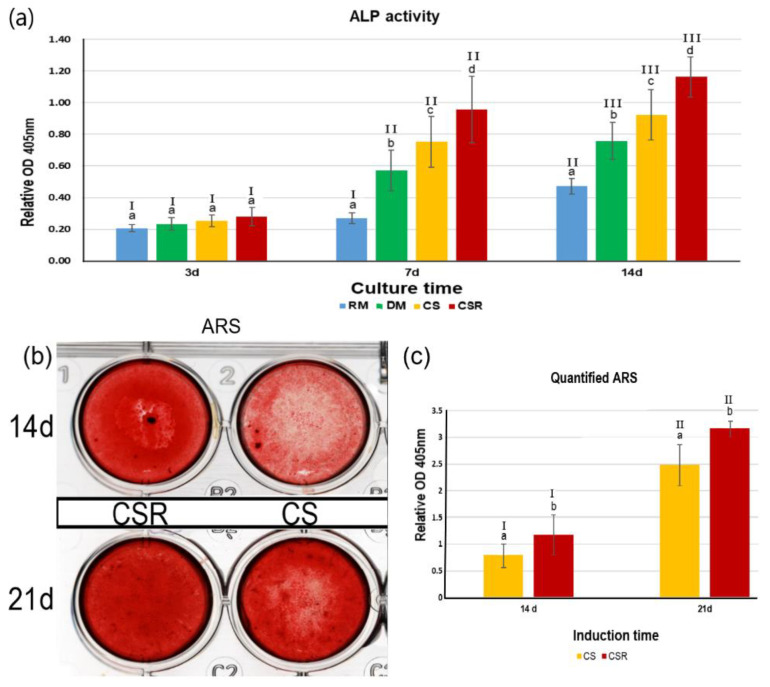
(**a**). ALP activity of CS and CSR after culturing for 3 d, 7 d, and 14 d. Regular medium (RM) and differentiation medium (DM) served as negative and positive controls, respectively. Figure 7. (**b**). Scanned plates showing mineralization nodules stained by ARS in the induced cultures at 14 d and 21 d in both CS and CSR. ARS was more intense in CSR than CS at both induction time points. Quantification of the mineralized nodules by ARS revealed higher OD values in CSR than CS (**c**). Different lowercase letters indicate significant difference between groups within each induction time point, while Roman numbers I, II, III indicate difference between each group at different induction time points, *p* < 0.05.

In Figure 7a, ALP activity of the differentiated HDPSC after exposure to CS and CSR extracts has increased over incubation times. Two-way ANOVA analysis showed that both factor 1—“groups” and factor 2—“different time-points” were statistically significant (*p* < 0.05). The interaction between groups and different culturing time points was also significant (*p* < 0.05). After 3 days of incubation, no significant difference was found between the groups (*p* > 0.05). After 7 days and 14 days of culture, the ALP activity of CSR was significantly higher than CS (*p* < 0.05). The ALP activity of CSR at different culture time points was statistically significant (*p* < 0.05). Furthermore, the ALP activity of CS was statistically significant at different culture time points (*p* < 0.05).

#### 3.8.2. Alizarin Red Staining

Results of ARS have shown that ARS was more intensive in the CSR group than in the CS group after 14 days and 21 days of culturing times, indicating more calcium deposits and mineralization nodules in the CSR group Figure 7b. Two-way ANOVA analysis of the quantified absorbed ARS revealed that factor 1— “groups” and factor 2— “time-points” were statistically significant (*p* < 0.05). While the interaction between the two factors was statistically non-significant (*p* = 0.066). The relative ARS OD was significantly higher in the CSR group than the CS group at both 14- and 21-day culturing times (*p* < 0.05), which was consistent with the qualitative results. The quantified ARS of CSR at 21 d culture was significantly higher than at 14 d culture (*p* < 0.05). Similarly, the quantified ARS of CS was significantly higher at the 21 d culture than the 14 d culture (Figure 7c).

### 3.9. ICP-OES

**Figure 8:**
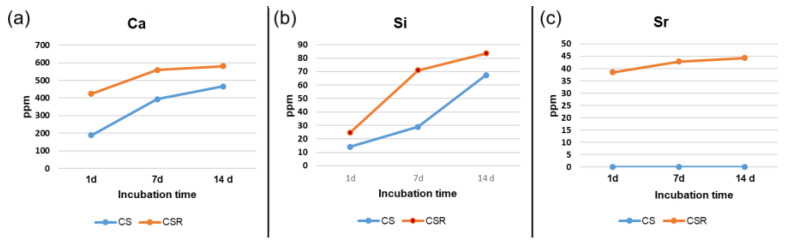
Ion release of Ca (**a**), Si (**b**), and Sr (**c**) in both CS and CSR using ICP-OES. The ion release of Ca, and Si was higher in CSR than in CS with gradual increase with time in all groups. Sr release also increased with immersion time in CSR. No Sr was detected in CS.

ICP-OES results revealed that the Ca and Si ions concentrations in CSR extracts were higher than those of CS extracts Figure 8a,b. The Sr ion concentration in the CSR group extracts was in the 45 ppm range, whereas no trace of Sr was found in the CS extracts Figure 8c.

## 4. Discussion

The present study developed a novel nanocrystalline calcium strontium silicate biocement (CaSrSiO_4_) via the sol–gel process. The synthesized cement demonstrated self-setting property with enhanced in vitro mineralization, ALP activity, and differentiation of HDPSC, surpassing CS. Thus, the null hypothesis tested was rejected. 

The potential of bioactive materials to interact with the surrounding tissues, and stimulate cellular differentiation and mineralized hard tissue deposition, has attracted increasing interest from researchers in dental and biomedical fields [1,33,34]. Moreover, there has been a rising demand for the preservation of pulp vitality and stimulation of dentin bridge formation, as in the case of direct pulp capping [35]. Furthermore, such bioactive materials are essential for the success of regenerative endodontics [36,37]. This substantiates the urge for optimizing calcium silicate-based cements to provide the best conditions for the attachment, proliferation, and differentiation of HDPSC.

Strontium has gained much attention in recent years, owing to its ability to stimulate cellular proliferation and osteogenesis and downregulate osteoclastic activity [25] with negligible systemic toxicity [16]. The present study demonstrated for the first time the enhanced stimulatory effect of calcium strontium silicate cement on the differentiation of HDPSC.

The XRD results revealed that after the incorporation of Sr, the phase composition of CS has changed, indicating the formation of (CaSrSiO_4_) with a symmetric orthorhombic crystalline structure. Our findings are the first to report the formation of CaSrSiO_4_ crystalline phase via the sol–gel method, unlike other studies which incorporated Sr into different calcium silicate compositions with no change in the calcium silicate main crystalline structure [17,25,38]. It is suggested that the high Sr content and sintering process have led to the change in the crystalline phase of CS and the formation of CaSrSiO_4_. Strontium is a similar divalent cation as calcium, and the radius of Sr is slightly bigger than Ca, thus strontium can replace some of the Ca in the calcium silicate structure [39]. 

Setting time plays a pivotal role in materials application in dental practice. CSR presented a self-setting property, indicating that CSR maintained similar hydration properties and the formation of calcium silicate hydrate as CS. Both cements exhibited short setting times, which is mostly owing to the porous structure of the powders and smaller particle size arising from the sol–gel synthesis [10,40]. The nanosized CSR powder had an average particle size of 86 nm as indicated by the ZetaView analyzer. The initial and final setting times of CSR were shorter than CS. Though the reason is yet unclear, it could be due to the interruption of the calcium silicate network by Sr, which allowed for the higher release of Ca with the subsequent rapid formation of calcium silicate hydrate after mixing with distilled water [25,41]. Liu et al. [42] reported in their study that the setting time of 0.5 mol% concentrations of Sr-substituted tri-calcium silicate was decreased, which supports our findings. In another study conducted by Huang et al. [25], who incorporated Sr into calcium silicate bone cement and concluded that the setting time was increased. However, the powder was synthesized by solid-state sintering, which produces powders with lower porosity than powders synthesized by the sol–gel process. This might slow down the hydration process [10]. CSR exhibited similar compressive strength to CS with no statistically significant difference.

The pH values of CSR were significantly lower than CS. This could be explained by the reduced formation of calcium hydroxide (a byproduct of the hydration reaction) in CSR compared to CS, which is the main contributor to the rise in pH of calcium silicate hydraulic cements. It has been reported that highly alkaline pH impairs cellular proliferation and growth [9]. Interestingly, the pH value of CSR was lower than CS, providing a better environment for cellular proliferation.

The deposition of an apatite layer on the ceramic surface in vitro reflects its ability to be bioactive in vivo. An apatite layer was formed on both CS and CSR, which increased with increasing immersion time in PBS. It was observed from SEM images that CSR showed higher apatite formation on its surface than CS at all immersion periods. We observed that the apatite-like crystal showed a spherical shape in CSR, especially after 7 days of immersion, which might be due to the release of Sr that replaced Ca in the apatite network [43]. The higher release of Ca in CSR by the effect of Sr would also explain the increased formation of apatite-like crystals in CSR [44]. Furthermore, Sr competes with Ca in the hydroxyapatite network, resulting in the formation of Sr-apatite [45]. The formed apatite layer on both cements was also characterized by FTIR analysis, which showed a band split at around 525–650 cm^−1^ arising from bending modes of P-O bonds, and at 1070 cm^−1^, which is due to the symmetric stretching vibration of PO4-3 [46], suggesting the high possibility of a well crystalline HAP.

To further investigate the bioactivity of CSR and its effect on the differentiation potential of HDPSC, ALP activity and mineralized nodule deposition by ARS were also evaluated. The differentiation of HDPSC into functional mineralizing linage is important for promoting dentinogenesis and successful pulp regeneration. ALP activity is an early marker of cellular differentiation and is correlated with the mineralization process [47]. HDPSC expressed higher early ALP activity in CSR after 3 days of culture, which was significantly higher than CS after 7 and 14 days. The mechanism of increased ALP in CSR is suggested to be due to the release of Sr ions, which have the affinity to bind to certain metal binding sites on ALP and enhance its activity [48]. Our findings agreed with other studies which reported that Sr enhanced ALP activity and differentiation of various multipotent stem cells, including DPSC [25,49]. The Sr ions that are released can increase ALP activity as well as osteoblast-related gene expression. Huang et al. [16] reported that SrCl_2_.6H_2_O stimulated the differentiation of HDPSC when incorporated into the culture medium and enhanced the ALP activity similar to osteoblastic differentiation, which is consistent with our findings. The mineralization in CSR was also significantly higher than CS after 14 and 21 days of culture, as indicated by ARS analysis. The increased calcium deposition indicates the enhanced differentiation effect of CSR on HDPSC. This enhanced differentiation is through the regulation of Wnt/β-catenin signaling pathway triggered by the released Sr ions [16,50].

The currently investigated cement could be a promising material for improving the prognosis of regenerative dental treatments, maintaining the vitality of dental pulp tissue, and stimulating new dentin formation. Referring to the limitations of the present study, since this is an in vitro study, further in vivo animal studies are required to validate and substantiate the enhanced in vitro performance of the developed CSR in terms of inflammatory pulpal response, calcified dentin bridge formation, and long-term biocompatibility and safety. However, the in vitro bioactivity, biocompatibility, and differentiation experiments are considered prime indicators of the expected performance of the developed cement in vivo. Future work will be directed to investigating the effect of CSR on the expression of genes related to odontogenic differentiation of HDPSC and the immunomodulatory potential of CSR.

## 5. Conclusions

Within the limitations of the present study, it can be concluded that calcium strontium silicate was successfully synthesized by the sol–gel process. CSR enhanced the differentiation of HDPSC into mineralizing lineage and demonstrated a shorter setting time than CS. Calcium strontium silicate outperformed conventional calcium silicate in terms of cytocompatibility and in vitro bioactivity. The compressive strength of CSR was similar to that of CS. Calcium strontium silicate is a promising material for potential dental applications.

## Figures and Tables

**Table 1:** pH values.

**Groups**	Initial pH	24 h	72 h
**Control**	6.9 ± 0.06 aI	7 ± 0.11 aI	7 ± 0.12 aI
**CS**	11.1 ± 0.12 bI	11.7 ± 0.21 bII	11.8 ± 0.16 bII
**CSR**	9.5 ± 0.15 cI	11.4 ± 0.07 cII	11.5 ± 0.14 cII

pH values of control, CS, and CSR immediately after setting (initial), after 24 h, and after 72 h. Lowercase letters a, b, c show significant differences between groups within each time point *p* < 0.05, While Roman numbers I, II, show significant differences within each group at different time points *p* < 0.05.

## Data Availability

The data presented in the study are available on request from the corresponding author.

## Abbreviations

ALP	Alkaline phosphatase
ANOVA	Analysis of variance
ARS	Alizarin red stain
CS	Calcium silicate
CSR	Calcium strontium silicate
DIW	Deionized water
DM	Differentiation medium
DMEM	Dulbecco Modified Eagle Medium
FTIR	Fourier-transformed infrared
HDPSC	Human dental pulp stem cells
ICP-OES	Inductively coupled plasma-optical emission spectrometry
MTT	3-(4,5-dimethylthiazol-2-yl)-2,5-diphenyltetrazolium bromide
OD	Optical density
RM	Regular medium
XRD	X-ray diffractometer
α-MEM	α-minimal essential medium
